# Risk factors associated with intermediate and long-term mortality following vascular surgery in South African patients

**DOI:** 10.5830/CVJA-2010-016

**Published:** 2010

**Authors:** BM Biccard, S Nepaul

**Affiliations:** Department of Anaesthetics, Nelson R Mandela School of Medicine, and Inkosi Albert Luthuli Central Hospital, Durban, South Africa; Department of Clinical Technology, Durban University of Technology, Durban, South Africa

## Abstract

**Summary:**

There are few data on predictors of mortality following vascular surgery in South African patients. While in the developed world, peri-operative risk factors are also associated with intermediate-term survival, it is likely that the weighting and even the clinical risk factors may be different in South African patients due to the epidemiology of cardiovascular disease in developing countries. The aim of this study was therefore to determine risk factors associated with intermediate and long-term mortality in South African vascular surgical patients.

**Design:**

A retrospective cohort study was conducted. Intermediate and long-term survival was determined by subsequent hospital visits or admissions. For patients who did not return to the hospital, the patient or patient’s next of kin was contacted telephonically. The outcome of the patient, and the time to the outcome following the surgical procedure were recorded. Bivariate and multivariate analysis was conducted using Cox regression analysis to determine predictors of intermediate-term mortality.

**Results:**

Only hypertension and diabetes were associated with intermediate and long-term mortality at the bivariate level of analysis with *p* < 0.10. There was no co-linearity between hypertension and diabetes. Hypertension was the only predictor of intermediate and long-term survival retained in the multivariate model (hazard ratio 3.86, 95% confidence interval 0.83–15.4, *p* = 0.086).

**Conclusion:**

In contrast to developed-world observations, peri-operative clinical risk indices were not associated with intermediate and long-term survival in South African vascular surgical patients. Instead, two risk factors that were identified in the South African National Burden of Disease study were associated with mortality. It appears that a ‘western lifestyle’ (and the presence of associated risk factors) may be more important predictors of intermediate and long-term mortality than peri-operative risk predictors of cardiac events in South African vascular surgical patients. This study highlights an important public health issue for the South African population where the most important determinants of mortality are continued exposure to risk factors (such as hypertension and diabetes) in the community, with little modification of these risk factors through primary health surveillance and management, even after surgical admission for pathology known to be associated with these risk factors.

## Summary

There is a paucity of data on predictors of cardiac mortality and all-cause mortality following vascular surgery in South African patients. This is a concern that the weighting and even the clinical risk factors may be different in South African patients in comparison to developed-world patients.^1^ In an attempt to address this situation, we have used an established vascular surgical database at Inkosi Albert Luthuli Central Hospital (IALCH) to determine risk factors associated with in-hospital cardiac^2^ and all-cause mortality.^3^

There are few data of intermediate (less than one year following surgery)^4^ and long-term (more than one year following surgery)^4^ outcomes following vascular surgery in South African patients. A study of femoral–distal bypass conducted between 1999 and 2002 in Cape Town showed a two-year mortality of 19.2%, but was too small to determine independent predictors of mortality.^5^

A long-term model of all-cause mortality following peripheral arterial surgery has been developed in a first-world population (Rotterdam study).^6^ Listed in decreasing order of importance, this study showed that one-year mortality was associated with an age above 65 years, renal dysfunction, hypercholesterolaemia, a history of congestive heart failure, an ankle-brachial index of < 0.60, Q-waves on ECG and diabetes.^6^

Therefore, five of the six clinical risk factors of Lee’s Revised Cardiac Risk Index (which are independent predictors of peri-operative cardiac events following non-cardiac surgery)^7^ were also independent predictors of one-year mortality.^6^ Indeed, the five-year mortality model^6^ included all of Lee’s clinical risk predictors.^7^ Therefore in a developed-world population, predictors of peri-operative cardiac events also appear to be predictors of intermediate and long-term mortality following vascular surgery.

The Rotterdam study is, however, probably of limited use in the South African population. This may be reflected in the difference in survival rates between the two studies. The one- and five- year mortality rate was 6 and 22%, respectively, in the Rotterdam study,^6^ which is lower than that reported in the Cape Town study of 19% at two years.^5^

The difference in long-term outcome reported between the Cape Town and Rotterdam studies may reflect differences in the epidemiological transition of cardiovascular disease,^8^ including socio-economic factors and exposure to risk factors, health surveillance and access to healthcare between a developed and a developing world population. Secondly, although beta-blockers, statins and aspirin were shown to improve long-term survival in the Rotterdam study,^6^ we know that the medical therapy of South African vascular patients is wholly inadequate.^2^,^3^

Determination of predictors of intermediate and long-term survival is important. These predictors may identify patients who require further aggressive risk-factor modification, therapy and increased surveillance postoperatively. In some cases, these risk predictors may even identify patients in whom conservative non-surgical management is preferable.^9^

The aim of this study was therefore to evaluate whether the clinical risk predictors identified in the Revised Cardiac Risk Index^7^ and our own studies of in-hospital cardiac^2^ and all-cause mortality^3^ were associated with intermediate and long-term mortality in South African patients who underwent elective or urgent vascular surgery. We also examined hypertension as a predictor of mortality as it is the second highest ranked risk factor associated with all-cause mortality in South Africans (following unsafe sex/sexually transmitted infections).^10^

## Methods

The Ethics Committee of the Nelson R Mandela School of Medicine for this study granted ethical approval. The patient cohort included all vascular surgical patients over 39 years of age admitted for both elective and emergency vascular surgery at Inkosi Albert Luthuli Central Hospital (IALCH) between June 2003 and June 2007.

From the hospital’s computerised database, we identified all patients who survived the surgical procedure and were discharged from hospital. To determine intermediate and longterm survival, all subsequent hospital clinic visits or hospital admissions were identified. For patients who did not return to the hospital, one of the authors (SN) used the registered contact details on the hospital database to contact the patient and/or the next of kin. The outcome of the patient (death or survival) and the time to the outcome following the surgical procedure were recorded.

The time to the outcome was grouped in six-month blocks. In presenting survival time, the six-month blocks were treated as a continuous variable, after confirmation that the distribution of the six-month blocks was of a normal distribution. Therefore, for example, a survival time of eight six-month blocks represents 48 months. ‘Lost to follow up’ was defined as a patient who, following discharge, had no further visit or admission to IALCH and neither the patient nor the next of kin were contactable using the contact details registered on the hospital database.

For all patients in whom we had intermediate and long-term outcome data, we extracted demographic data associated with peri-operative cardiac risk,^7^ and intra-hospital cardiac^2^ and all-cause mortality.^3^ Data on the following clinical risk factors were collected: history of ischaemic heart disease (or pathological Q waves on ECG), history of congestive heart failure, diabetes, serum creatinine > 180 µmol.l^–1^, history of cerebrovascular accident, age, gender, history of smoking, and history of hypertension.^2^,^3^,^7^,^11^

Data on medical therapy collected included chronic pre-operoperative statin therapy, beta-blocker therapy and postoperative beta-blocker withdrawal.^3^ Data on the surgical procedure included major vascular surgery, and out-of-hours surgery.^3^ Physiological data collected included the mean daily heart rate on the day before surgery and the third postoperative day, and whether the mean systolic blood pressure (SBP) was < 100 or > 179 mmHg on the third postoperative day.^3^

## Statistical analyses

To compare survivors and non-survivors, all categorical data were analysed using descriptive statistics and either the Fisher’s exact test or Pearson’s Chi-square test, where appropriate. All continuous data were analysed using descriptive statistics and compared using independent samples *t*-test, as all continuous data were normally distributed.

Bivariate and multivariate analysis was conducted using Cox regression analysis to determine predictors of intermediate and long-term mortality. Cases with missing data were excluded from the analysis. Risk factors with *p* < 0.10 on bivariate analysis were entered into the multivariate regression analysis. A backward stepwise modelling technique was used, based on likelihood ratios with entry and removal probabilities set at 0.05 and 0.1, respectively.

Co-linearity was also investigated. Co-linearity was considered if Pearson’s correlation coefficient was > 0.6 or the standard error of a covariate was > 5.0.^12^ If co-linearity was identified, the multivariate analysis was repeated after removal of the responsible covariate.^13^

Kaplan-Meier survival plots analyses were conducted for risk factors associated with *p* < 0.10 for bivariate Cox regression analysis. Both the log-rank and Breslow tests are reported.

The hazard ratio (HR) for intermediate and long-term death rates and 95% confidence intervals (CI) are reported. SPSS 15.0 for Windows (6 Sept 2006) was used for data analysis.

## Results

Over the four-year period, a total of 747 patients over the age of 39 years were discharged from hospital following successful vascular surgery. Four hundred and sixty-four patients were lost to follow up; therefore 283 patients were included in this study. There were 21 intermediate and long-term non-survivors and 262 survivors. The demographics and clinical, surgical and physiological risk factors are presented in Table 1. The data set was complete for all the risk factors listed in Table 1 with the exception of the serum creatinine in 20 patients (7.1%) and the mean daily heart rate on the third postoperative day in six patients (2.1%).

**Table 1.** Clinical Risk Factors And Intermediate And Long-Term Mortality Following Vascular Surgery

*Clinical risk factors*	*Non-survivors*	*Survivors*	p*-value*
Pre-operative risk factors			
Male gender	12/21 (57%)	170/262 (65%)	0.49^†^
Age	64.2	62.4	0.42
History of smoking	12/21 (57%)	162/262 (62%)	0.65^†^
Ischaemic heart disease	14/21 (67%)	174/262 (66%)	1.00^†^
Congestive cardiac failure	0/21 (0%)	6/262 (2.3%)	0.48^*^
Cerebrovascular accident	7/21 (33%)	80/262 (31%)	0.81^†^
Diabetes	13/21 (62%)	111/262 (42%)	0.11^†^
Hypertension	19/21 (91%)	192/262 (73%)	0.12^†^
Creatinine > 180 μmol.l^-1 ‡^	2/21 (9.5%)	13/242 (5.4%)	0.43^*^
Chronic medical therapy			
Pre-operative chronic beta-blockade	6/21 (29%)	80/262 (31%)	1.00^†^
Pre-operative statin therapy	5/21 (24%)	61/262 (23%)	1.00^†^
Mean daily HR day before surgery	73	75	0.42
Major vascular surgery	13/21 (62%)	155/262 (59%)	1.00^†^
Mean daily HR day 3 postop	81	82	0.65

HR: heart rate; *Pearson Chi-square test, ^†^Fisher’s Exact test; ^‡^data missing for 20 cases.

The bivariate Cox regression analysis of survival is presented in Table 2. Only hypertension and diabetes were associated with intermediate and long-term mortality at the bivariate level of analysis with *p* < 0.10. There was no co-linearity between hypertension and diabetes. Entering hypertension and diabetes into a multivariate Cox regression analysis resulted in hypertension being the only predictor of intermediate and long-term survival (HR 3.86, 95% CI: 0.83–15.4, *p* = 0.086) retained in the model.

**Table 2.** Bivariate COX Regression Analysis Of Predictors Of Intermediate And Long-Term All-Cause Mortality Following Vascular Surgery In Patients ≥ 40 Years Of Age

*Characteristic*	*Crude hazard ratio*	*95% CI*	p*-value*
Pre-operative risk factors			
Male gender	0.77	0.32–1.82	0.55
Age	1.02	0.97–1.07	0.40
History of smoking	0.96	0.40–2.28	0.92
Ischaemic heart disease	1.05	0.42–2.61	0.91
Congestive cardiac failure	0.05	0.0–89.0	0.68
Cerebrovascular accident	1.19	0.48–2.94	0.71
Diabetes	2.28	0.94–5.52	0.07
Hypertension	3.59	0.83–15.4	0.09
Creatinine > 180 μmol.l^-1^	1.87	0.43–8.06	0.40
Chronic medical therapy			
Pre-operative chronic beta-blockade	0.83	0.32–2.13	0.69
Pre-operative statin therapy	0.94	0.34–2.56	0.90
Withdrawal of chronic beta-blockade	1.11	0.37–3.30	0.86
Surgical risk factors			
Major vascular surgery	1.05	0.44–2.54	0.91
Surgery out of hours	0.77	0.10–5.75	0.80
Physiological data			
Mean daily HR day before surgery	0.98	0.95–1.02	0.37
Mean daily HR day 3 postop	1.0	0.97–1.02	0.52
Mean daily SBP < 100 or > 179 mmHg	2.09	0.61–7.08	0.24

CI: confidence interval; SBP: systolic blood pressure; HR: heart rate.

**Fig. 1.**
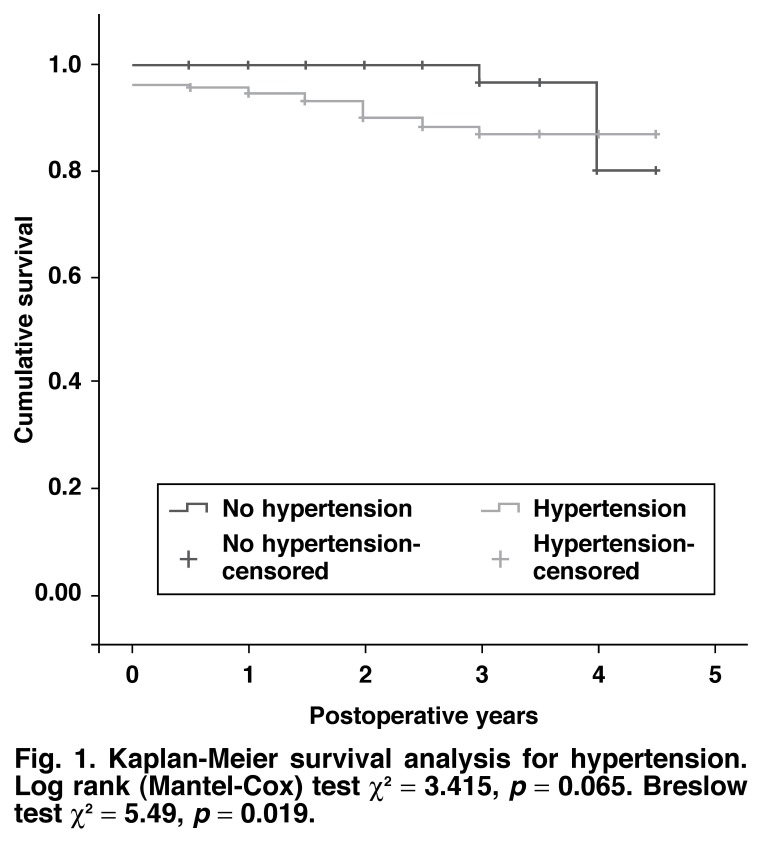
Kaplan-Meier survival analysis for hypertension. Log rank (Mantel-Cox) test χ^2^ = 3.415, *p* = 0.065. Breslow test χ^2^ = 5.49, *p* = 0.019.

**Fig. 2.**
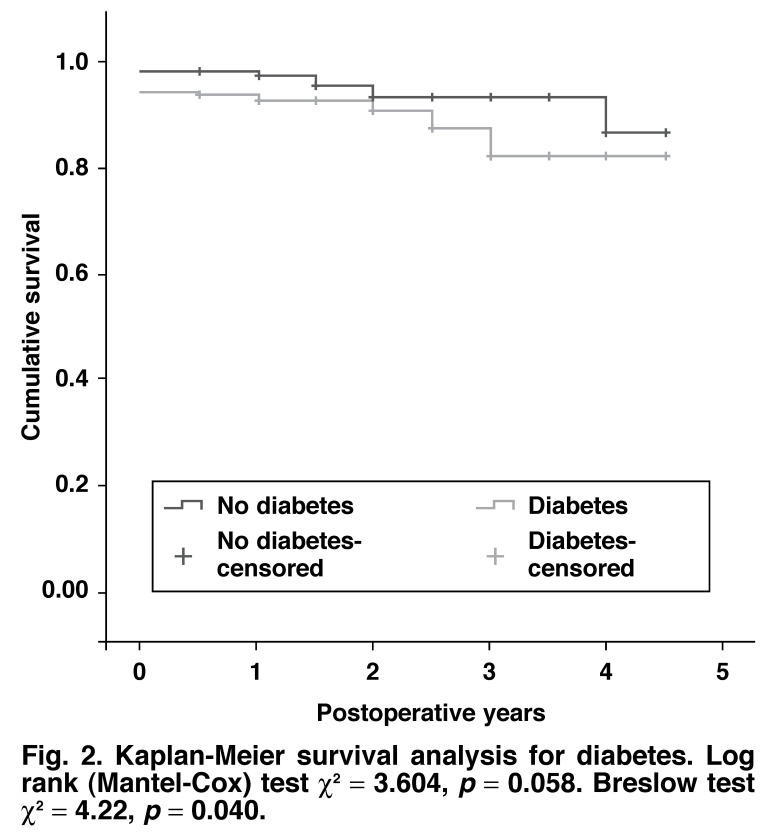
Kaplan-Meier survival analysis for diabetes. Log rank (Mantel-Cox) test χ^2^ = 3.604, *p* = 0.058. Breslow test χ^2^ = 4.22, *p* = 0.040.

The Kaplan-Meier survival curves for hypertension and diabetes are shown in Figs 1 and 2. The survival characteristics of patients with hypertension and diabetes are shown in Table 3.

**Table 3.** Survival Characteristics Of Patients With Hypertension And Diabetes

*Group*	*Cases* (n)	*Events* (n)	*Censored* (n)	Mean survival time in 6-month blocks (95% CI)*
No hypertension	72	2	70	8.73 (8.4–9.1)
Hypertension	211	19	192	8.18 (7.8–8.5)
No diabetes	159	8	151	8.55 (8.2–8.9)
Diabetes	124	13	111	8.04 (7.6–8.5)

*Survival time in 6-month blocks; normal distribution with a skewness statistic of 0.315.

## Discussion

This article highlights a number of important South African public health issues. The prevalence of clinical risk predictors in South African vascular patients was similar to or higher than that reported in European vascular patients^6^ (Table 4), with the exception of congestive cardiac failure and male gender. This is consistent with our understanding of epidemiological transition in a developing country,^8^ and an increasing burden of cardiovascular risk factors.

**Table 4.** Prevalence Of Clinical Risk Factors In South African And Dutch Vascular Patients

*Clinical risk factor*	*South African patients (n = 283)*	*Rotterdam study^6^ (n = 1332)*	p*-value*
Male gender	182 (64%)	964 (72%)	0.007
Ischaemic heart disease	188 (66%)	601 (45%)	< 0.001
Congestive cardiac failure	6 (2%)	105 (8%)	< 0.001
Cerebrovascular accident	87 (31%)	101 (8%)	< 0.001
Diabetes	124 (44%)	229 (17%)	< 0.001
Hypertension	209 (74%)	609 (46%)	< 0.001
Serum creatinine > 180 μmol.l^-1^	15 (6%)^*^	67 (5%)^†^	0.88
Pre-operative beta-blockers	86 (30%)	335 (25%)	0.07
Pre-operative statins	66 (20%)	257 (19%)	0.17

*Denominator 263 patients; ^†^renal dysfunction.

This study allows us to evaluate the relative importance of clinical risk factors, chronic medical therapy, surgical procedural risk factors and peri-operative physiological data on intermediate and long-term survival following vascular surgery. The Rotterdam study suggested that clinical risk factors associated with peri-operative cardiac events^6^,^7^ are also predictive of longterm mortality following peripheral vascular surgery. Our study could not confirm this observation in South African patients. Indeed, hypertension and diabetes (the two bivariate predictors of intermediate and long-term mortality identified in our patients) are not consistently associated with an adverse cardiac outcome in the peri-operative literature.^7^,^14^ Diabetes was not significantly associated with mortality in the only risk index of vascular surgical patients,^14^ and it was not significantly associated with major cardiovascular complications in the validation cohort of the Revised Cardiac Risk Index.^7^

A meta-analysis of hypertension in peri-operative patients suggests that it is statistically associated with cardiac morbidity, although the clinical importance of this finding is more difficult to quantify.^15^ Hypertension is not conventionally used to stratify cardiac risk in non-cardiac surgery.^16^ Similarly, in the Rotterdam study, hypertension was not identified as a predictor of mortality at one, five or 10 years following peripheral vascular surgery.^6^

Although hypertension and diabetes are either not important or inconsistently important risk predictors of mortality following vascular surgery in European and American populations, they were identified as important predictors of mortality in the South African National Burden of Disease study.^10^ This study identified three broad risk categories: mortality associated with sexually transmitted diseases, poverty and a western lifestyle. Risk factors associated with a western lifestyle included hypertension (second), tobacco smoking (third), high body mass index (fifth), high cholesterol (seventh), diabetes (eighth), physical inactivity (ninth) and low fruit and vegetable intake (tenth).

It is possible that South African vascular surgical patients returning to a western lifestyle in the community (and the presence of associated risk factors) may be a more important predictor of intermediate and long-term mortality than the established clinical risk factors associated with peri-operative cardiac risk,^6^,^7^ which also appear to be important predictors of intermediate and long-term survival in developed-world patients.

If this is true, this study highlights an important public health issue for a South African population in epidemiological transition, where the most important determinants of mortality are continued exposure to a risk factor (such as hypertension and diabetes) with little modification of these risk factors through health surveillance and management. It is likely that to improve survival in South African vascular surgical patients, a concerted public health initiative is necessary. Community-based riskfactor modification, surveillance and therapy should be considered of paramount importance.

The large number of patients ‘lost to follow up’ is also indicative of a dysfunctional primary healthcare system. Vascular surgical patients are patients who would benefit from continuing risk-factor modification and surveillance. Indeed, chronic medical therapy including statins, beta-blockers and angiotensin converting inhibitors are associated with improved survival following peripheral vascular surgery.^17^

Our study could not confirm the efficacy of cardiac medical therapy in South African vascular surgical patients because it was underpowered. Based on an HR of 0.68 for long-term mortality following peripheral vascular surgery associated with beta-blocker therapy,^17^ a study of over 3 100 patients would be required with a control event rate of 7.4%.^18^

Although, there is increasing evidence that physiological data may be predictive of intermediate and long-term survival following major and intermediate-risk non-cardiac surgery,^19^ this study could not confirm this. The sample size in this study may have been too small to show this association.

Our study had two limitations. Firstly, it was a retrospective study, and therefore it is possible that not all the risk predictors were recorded in the pre-operative medical charts.

Secondly, this study was limited by its sample size. It is likely that physiological data may still be predictive of intermediate and long-term survival and that chronic cardiac medication may improve long-term survival. It is however, the largest study we are aware of that has attempted to determine predictors of intermediate and long-term mortality in South African patients.

Despite these study limitations, it appears that traditional public health issues are more predictive of mortality for South African vascular surgical patients than internationally accepted peri-operative risk indices of cardiac morbidity and mortality, which are predictive in developed-world patients. These findings suggest that risk predictors for mortality are not necessarily the same in South African patients, when compared with European and American patients. It is therefore imperative that we continue to identify clinical predictors in South African patients, as it is likely that they are different to those published in the international literature.

## Conclusions

In contrast to developed-world observations, peri-operative clinical risk indices were not associated with intermediate and long-term survival in South African vascular surgical patients. Instead, hypertension was the only predictor of intermediate and long-term survival retained in the multivariate model, which has also previously been identified as the second most important predictor of mortality in the South African National Burden of Disease study.^10^
